# MiR‐144‐induced KLF2 inhibition and NF‐kappaB/CXCR1 activation promote neutrophil extracellular trap–induced transfusion‐related acute lung injury

**DOI:** 10.1111/jcmm.16650

**Published:** 2021-06-13

**Authors:** Aiping Le, Yize Wu, Wei Liu, Chenggao Wu, Piaoping Hu, Juan Zou, Linju Kuang

**Affiliations:** ^1^ Departments of Blood Transfusion The First Affiliated Hospital of Nanchang University Nanchang China

**Keywords:** kruppel‐like factor 2, microRNA‐144, neutrophil extracellular trap, nuclear factor kappa‐B/chemokine (C‐X‐C motif) receptor 1 signalling pathway, transfusion‐related acute lung injury

## Abstract

Transfusion‐related acute lung injury (TRALI) is a clinical syndrome which is associated with the formation of neutrophil extracellular trap (NET). Recent studies have demonstrated the roles of microRNAs (miRNAs) in the pathophysiological process of TRALI. Here, the study focused on the role of miR‐144 and the molecular mechanisms in NET‐induced TRALI. Up‐regulated miR‐144 and under‐expressed KLF2 were determined in patients with TRALI. In the mouse model of a two‐event TRALI induced by intraperitoneal injections with lipopolysaccharide and anti‐H‐2Kd mAb, we determined expression patterns of miR‐144, Krüppel‐like factor 2 (KLF2), chemokine (C‐X‐C motif) receptor 1 (CXCR1) and nuclear factor kappa‐B (NF‐kappaB) p65. The results confirmed that miR‐144 was highly expressed, while KLF2 was poorly expressed in mice with TRALI. Dual‐luciferase reporter gene assay identified that miR‐144 could target KLF2. Using gain‐ and loss‐of‐function approaches, we analysed the effects of miR‐144 and its interaction with KLF2 on TRALI. Enforced expression of miR‐144 was found to aggravate NET‐induced TRALI by down‐regulating KLF2 and activating the NF‐kappaB/CXCR1 signalling pathway in TRALI mice. Collectively, miR‐144‐targeted inhibition of KLF2 and activation of NF‐kappaB/CXCR1 are possible mechanisms responsible for NET‐caused TRALI. These findings aid in the development of therapeutic modalities for the treatment of TRALI.

## 1 INTRODUCTION

Transfusion‐related acute lung injury (TRALI) represents a clinical syndrome that occurred within a few hours after the transfusion of blood or blood products, with acute non‐cardiogenic pulmonary oedema and hypoxaemia as the main manifestations.^1^ Definitions of TRALI have evolved over time with most of them incorporating all or many facets of expert consensus, and the well‐recognized ‘classic’ definition of TRALI includes the following requirements: oxygen saturation <90% and Pao2/Fio2 <300 along with symptoms of hypoxia, including bi‐pulmonary infiltration detected by X‐ray.^2^ It should also be noted that a redefinition of TRALI has been developed recently based on modifications of the commonly used 2004 TRALI Canadian Consensus Conference definition. Specifically, TRALI has been separated into two types: TRALI type I applies to cases without an risk of acute respiratory distress syndrome (ARDS) and TRALI type II to cases with mild pre‐existing ARDS or ARDS risk factors.^3^

According to statistics from a recent report, TRALI has become one of the leading causes of death due to blood transfusions.^4^ Presently, related studies have suggested that the pathogenesis of TRALI is primarily related to neutrophils.^5^ Therefore, it is of great significance to develop novel effective treatment modalities in managing TRALI. Previous researches have demonstrated the existence of a neutrophil extracellular trap (NET) in inflammatory injured lung tissues.^6^ NETs are extracellular network structures formed by neutrophil DNA, histones and granular proteins in the cytoplasm, usually combining into larger structures with a diameter approximately 50 nm.^7^ Excessive formation of NET in the body is associated with several pathological disease processes,^8^ such as systemic lupus erythematosus ^9^ and thrombosis.^10^ Therefore, the reduction of the excessive formation of NET is one of the methods for treating TRALI.^11^

Previous studies have uncovered the up‐regulated miR‐144 in acute lung injury.^12^, ^13^ The involvement of KLF2 in acute lung injuries has been identified in a variety of animal models.^14^ Moreover, studies have indicated that nuclear factor kappa‐B1 (NF‐κB1), one of the major genes of the NF‐κB pathway, can affect acute lung injury.^15^, ^16^, ^17^ NF‐κB can mediate the expression of chemokine (C‐X‐C motif) receptor 1 (CXCR1), as CXCR1 is a potential therapeutic target for acute lung injury.^18^, ^19^, ^20^, ^21^ More importantly, accumulating evidence on murine models of TRALI^22^, ^23^, ^24^ has suggested the feasibility of developing mouse models to investigate TRALI. Based on the aforementioned findings, we aimed to verify in the present study whether miR‐144/KLF2/NF‐κB/CXCR1 axis was involved in NET‐induced TRALI, in both clinically collected samples and mouse models of TRALI, which may help to understand the molecular mechanisms of NET‐induced TRALI.

## 2 MATERIALS AND METHODS

### 2.1 Ethical **statement**

This study was approved by the Ethics Committee of the First Affiliated Hospital of Nanchang University. All experiments were conducted in strict accordance with principles of the Helsinki Declaration. Written informed consents were signed and submitted by each participant. Animal experiments were performed with the approval of the Experimental Animal Ethics Committee of the First Affiliated Hospital of Nanchang University. All efforts were made to minimize the suffering of experimental animals.

### 2.2 Bioinformatics analysis

TRALI‐related microarray data set GSE11434 was retrieved from the Gene Expression Omnibus database (https://www.ncbi.nlm.nih.gov/gds). The expressions of TRALI‐related genes were analysed using R language with the box diagram obtained. An online analysis tool, String (https://string‐db.org), was then used to construct a protein‐protein interaction (PPI) network of TRALI‐related genes. The key downstream genes and the relevant pathways were confirmed based on existing literature.

### 2.3 Study subjects

Peripheral blood was collected from 10 healthy blood donors and 10 patients with TRALI from the First Affiliated Hospital of Nanchang University. Patients were diagnosed with TRALI based on Delphi panel method.^25^ All blood samples were centrifuged at 800 × *g* for 10 minutes to collect plasma and were subsequently stored at −80°C. Meanwhile, lung biopsy samples were collected from the 10 TRALI patients, none of which received anti‐tumour treatment before surgery. Lung biopsy samples from 10 non‐TRALI patients were also collected as controls. The samples are stored at −80°C for subsequent analysis.

### 2.4 Establishment of two‐event TRALI mouse models

A total of 160 male BALB/c mice, aged 8‐10 weeks and weighing 24‐28 g, were purchased from the Experimental Animal Center of The First Affiliated Hospital of Nanchang University. Then, 150 randomly selected mice were subjected to a two‐event TRALI model establishment as previously indicated.^26^ In brief, mice were injected intraperitoneally with lipopolysaccharide (LPS) (0.1 mg/kg) 24 hours before the introduction of anaesthesia through a retro‐orbital injection of xylazine (20 mg/kg) and ketamine (100 mg/kg) and treated with an anti‐H‐2Kd mAb (clone 34‐1‐2S, 0.5 mg/kg), its isotype control or saline. The mice were maintained at 37°C and killed after 10 minutes or 2 hours following the administration of the anti‐major histocompatibility complex (MHC) type I mAb. All procedures were conducted in a blinded manner. The mice were grouped and injected with different plasmids, activator or inhibitor for the NF‐κB signalling pathway, 48 hours before the injection of MHC I monoclonal antibody *via* the jugular vein. They were injected with miR‐144 mimic, miR‐144 inhibitor, overexpressed (oe)‐KLF2, miR‐144 mimic +oe‐KLF2 or their corresponding negative controls (NCs) (NC mimic, NC inhibitor, oe‐NC, miR‐144 mimic +oe‐NC, oe‐KLF2 + mimic NC, oe‐NC +mimic NC) *via* tail vein (n = 10). In the presence of miR‐144 mimic or oe‐KLF2, 1% pyrrolidine dithiocarbamate (PDTC, an inhibitor of the NF‐κB signalling pathway, 100 mg/kg) or 1% phorbol 12‐myristate 13‐acetate (PMA, an activator for the NF‐κB signalling pathway, 100 mg/kg) was injected intraperitoneally (n = 10). Dimethyl sulphoxide (DMSO) was intraperitoneally injected as NCs (DMSO, oe‐KLF2 + DMSO and miR‐144 mimic +DMSO) (n = 10). The final concentration of oe‐NC, oe‐KLF2, miR‐144 mimic, NC mimic, miR‐144 inhibitor and NC inhibitor was 50 nM.

### 2.5 Bronchoalveolar lavage fluid (BALF) collection

TRALI mice were killed for bleeding, and a 20‐gauge plastic catheter was inserted into the trachea, followed by three consecutive infusions of 1 mL of warm saline to flush the lungs. Collected BALF was then centrifuged, and cells were harvested and resuspended in PBS.

### 2.6 Flow cytometric detection of CD4^+^ cells

Cells collected from BALF were subjected to flow cytometry to determine the proportion of CD4^+^ cells. The cells were stained with anti‐CD4 PECy5 (DAKO, Diagnostics BV) for FACS analysis, and active CD4^+^ T cells were screened out utilizing forward scatter/side scatter (FSC/SSC). CellQuest software was then used to analyse the expression of cell markers, and the proportion of CD4^+^ cells was reflected by percentage of positive cells or mean fluorescence intensity (MFI).

### 2.7 Enzyme‐linked immunosorbent assay (ELISA)

Levels of IL‐6, IL‐8 and IL‐10 in BALF were measured utilizing ELISA kits for IL‐6 (ab242233), IL‐8 (ab242232) and IL‐10 (ab255729), respectively. Myeloperoxidase (MPO) antibodies (50 μL of 5 μg/mL, catalogue no. 07‐496, Upstate Technology) were added to each well in a 96‐well plate and incubated at 4°C overnight, followed by being washed 3 times with deionized water (300 μL each time). Afterwards, 80 μL of incubation buffer containing peroxidase‐labelled anti‐DNA monoclonal antibodies (Cell Death ELISAPLUS, Roche; 1:25) was added to each well. Further, 20 μL samples were added for a 2‐hour incubation period at room temperature. After 3 times of washing with deionized water (300 μL each time), each well was added with 100 μL of 2, 2'‐azino‐bis(3‐ethylbenzothiazoline‐6‐sulphonic acid), following 20 minutes of incubation in the dark at room temperature. Lastly, the absorbance value was measured at 405 nm and the final concentrations in each well were calculated according to the standard curve.

### 2.8 Extravascular lung water (EVLW) and extravascular plasma equivalents (EVPEs)

The extracted lungs were weighed, homogenized and then placed in an oven for 24 hours. EVLW was calculated by measuring the amount of haemoglobin in lung homogenate. In order to test the permeability of the pulmonary blood vessels to proteins, mice were perfused with 125i‐labelled albumin (Iso‐Tex Diagnostics Inc). Lastly, EVPEs were determined as the radioactivity ratio of blood to bloodless lung by utilizing a gamma counter (Packard 5000 series).

### 2.9 Identification of NET in mouse lung sections

Pathological sections of the lung tissue (7 µm thickness) were incubated for 60 minutes at room temperature with antibodies against MPO (ab45977, 1:500, Abcam) and histone (PA5‐40087, 1:500, Invitrogen), followed by incubating with fluorescein isothiocyanate–labelled goat anti‐rabbit secondary antibodies to immunoglobulin G (IgG) (ab6717, 1:500, Abcam) and cy3‐labelled goat anti‐rabbit secondary IgG (ab6939, 1:500, Abcam) in the dark. The sections were then stained with 4',6‐diamidino‐2‐phenylindole and observed with a laser scanning confocal microscope (LSM510, Carl Zeiss, Diagnostic Instruments Inc).

### 2.10 Lung wet/dry (W/D) weight ratio

Lung W/D weight ratios were measured to be indicative of pulmonary oedema. The right lung of each mouse was removed and weighed as the wet weight, then dried in an oven at 60°C for 48 hours and were reweighed as the dry weight. W/D ratio = the net wet weight/the net dry weight.

### 2.11 Neutrophil extraction

Neutrophils were extracted by natural sedimentation, in combination with a density gradient centrifugation. One h after modelling, the peripheral blood was collected and incubated at 37°C for 12 hours to allow the red blood cells to settle naturally. Then, 1 mL polymorphonuclear neutrophil separation solution was added into a 15‐mL centrifuge tube, followed by the slow addition of the upper plasma layer. After 8 minutes of low‐speed gradient centrifugation at 700 rpm, the liquid in the centrifuge tube was divided into four layers: the upper layer consisting of plasma and platelets, the middle white blood cells being the granulocyte layer, the lower layer as the separation solution and the bottom layer with red blood cells. A separation solution (1 mL) and the middle cell layer were added to the centrifuge tube for further use. After centrifuging the mixture at 2500 rpm for 2 minutes, the precipitates were neutrophils.

### 2.12 Dual‐luciferase reporter gene assay

The KLF2 3′ untranslated region (3′UTR) fragment, containing binding sites to miR‐144, was inserted into the pGL3 plasmid. By using the point mutation method, the KLF2‐3′UTR‐mutant (MUT) fragment containing mutated binding sites was constructed and inserted into the pGL3 plasmid. Through liposome transfection, the correctly sequenced pGL3‐KLF2‐wild‐type (WT) and pGL3‐KLF2‐MUT recombinant vector were cotransfected into HEK293T cells with either miR‐144 mimic or NC mimic. The cells were collected and lysed after being transfected for 48 hours, and the relative light unit of each sample was detected using a luciferase detection kit (K801‐200, Biovision) at dual‐luciferase reporter gene analysis system (Promega). The relative luminescence activity was determined as the ratio of the firefly luciferase relative light unit (RLU) to the Renilla luciferase RLU (internal reference).

### 2.13 Reverse transcription quantitative polymerase chain reaction (RT‐qPCR)

The total RNA content was extracted from tissues using TRIzol reagent (Cat. No. 16096020, Thermo Fisher Scientific). Then, 5 µg of total RNA was reversely transcribed into cDNA following the instructions of the cDNA Kit (K1622; Fermentas Inc). RT‐qPCR was then performed in compliance with the TaqMan Gene Expression Assays protocol (Applied Biosystems) with cDNA as a template. U6 served as an internal reference for miR‐144, while glyceraldehyde‐3‐phosphate dehydrogenase (GAPDH) served that for other genes. The primer used are shown in Supplementary Table 1. Relative quantification (2^‐△△CT^ method) was used to calculate the relative level of expression of the target gene.^27^

### 2.14 Western blot analysis

Cells were lysed with a lysis buffer containing phenylmethylsulphonyl fluoride on ice for 30 minutes. The supernatant was collected after centrifugation at 10 000 rpm at 4°C for 15 minutes. The protein concentration of each sample was determined using the bicinchoninic acid kit (Thermo Fisher Scientific). Then, 30 μg of the total protein contents were subjected to polyacrylamide gel electrophoresis and transferred onto a polyvinylidene fluoride membrane (Amersham). The membrane was blocked with 5% skimmed milk at room temperature for 1 hour and incubated at 4°C overnight with the following primary antibodies: rabbit polyclonal antibodies to KLF2 (ab17008, 1:1000, Abcam), phosphorylated‐NF‐κB p65 (ab194726, 1:1000, Abcam), CXCR1 (ab124344, 1:1000, Abcam) and GAPDH (ab9485, 1:2000, Abcam). The following day, the membrane was re‐probed with horseradish peroxidase–labelled secondary goat anti‐rabbit antibody IgG (ab6721, 1:2000, Abcam) for 1 hour at room temperature. After scanning and development by optical luminometer (General Electric Company), the protein bands were quantified by Image Pro Plus 6.0 software (Media Cybernetics).

### 2.15 Haematoxylin‐eosin (HE) staining

After the induction of anaesthesia, a thoracotomy procedure was performed on mice through perfusion using normal saline (4°C) and 4% paraformaldehyde, avoiding any injury on the lung. The lung tissues were later collected, fixed in 10% neutral buffered formalin overnight, embedded with paraffin and sliced into sections for the subsequent microscopic observation of cell morphology following HE staining. Briefly, sections were dewaxed in xylene for 2 times (5‐15 minutes/time), dehydrated in absolute alcohol (100%, 95%, 80% and 75%, 1 minute/time) and washed by running water for 3 minutes. Haematoxylin and eosin dyes were allowed to stain on the sections for 8 minutes and 2 minutes, respectively. After conventional dehydration and rinsing, sections were fixed in a neutral balsam and observed at a high magnification for cell morphological changes.

### 2.16 Immunohistochemistry

Paraffin‐embedded sections of lung tissues were deparaffinized and dehydrated, followed by antigen retrieval in Tris‐EDTA buffer solution (pH 9.0) under high heat for 5 minutes twice. Non‐specific proteins were blocked and washed 3 times with PBS solution for 3 minutes each time. Treated sections were labelled with a circle around the tissue, and 5% sheep serum was added into the circle, followed by 30‐minute incubation in a humid box at room temperature. Subsequently, the sections were incubated with primary antibody against KLF2 (diluted in PBS, PA5‐40591, 1:100, Thermo Fisher Scientific) for 1 hour in a humid box at 4ºC overnight and reheated at 37ºC for 45 minutes. Afterwards, the sections were incubated with secondary antibody (ab6712, 1:1000, Abcam) at 37ºC for 30 minutes. After washing, colour was incubated with DAB solution (200ul, Shgma) for 20 minutes in the dark for colour development, followed by counter‐staining with haematoxylin solution, dehydration, permeabilization and mounting.

### 2.17 FISH assay

Deparaffinized and dehydrated sections of lung tissues were treated with citrate (PH 6.0), boiled for 40 minutes and incubated with proteinase K at 37°C for 10 minutes. After fixing and dehydration, the sections were incubated with pre‐hybridization solution at 42°C for 1 hour and then with probe hybridization solution at 42°C overnight, followed by DAPI staining of the nucleus and microscopic observation.

### 2.18 Statistical analysis

SPSS 21.0 (IBM Corp.) software was used for the analysis of statistical data. Measurement data were expressed by the mean ± standard deviation. If data were in compliance with normal distribution and homogeneity, comparisons of data between two groups were performed by a paired *t* test, while an unpaired *t* test was used to compare those in unpaired design. Data comparisons among multiple groups were conducted using a one‐way analysis of variance (ANOVA), followed by Tukey's post hoc test. Data at different time‐points were compared using repeated measures of ANOVA, followed by Bonferroni's post hoc test. A Pearson correlation coefficient was used to analyse the correlation between the two indicators. Moreover, the Kaplan‐Meier method was used to calculate the survival rate of mice, and survival difference was analysed by a log‐rank test. *P* < .05 indicated the difference was statistically significant.

## 3 RESULTS

### 3.1 miR‐144 was highly expressed in blood samples of patients and mice with TRALI

**FIGURE 1:**
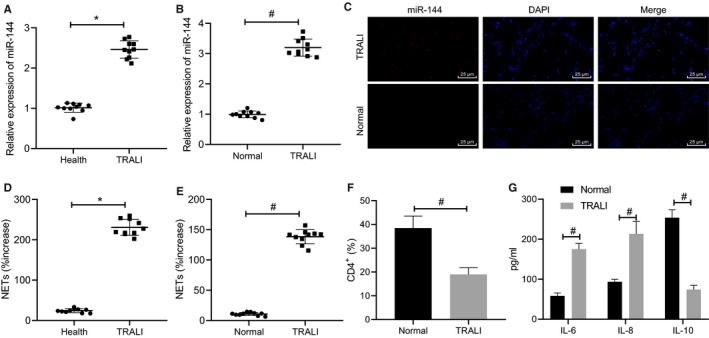
The high expression of miR‐144 in blood of patients and mice with TRALI. A, The expression of miR‐144 in blood samples of healthy donors and patients with TRALI, determined by RT‐qPCR. B, The expression of miR‐144 in blood samples of normal mice and TRALI mice evaluated by RT‐qPCR. C, FISH assay to measure miR‐144 level in lung tissues of patients with TRALI (×400). D, The formation of NET in blood samples of healthy donors and patients with TRALI detected by MPO‐DNA ELISA method. E, The formation of NET in blood samples of normal mice and TRALI mice detected by MPO‐DNA ELISA method. F, Flow cytometry to detect the proportion of CD4^+^ cells in BALF of mice. G, ELISA to measure levels of IL‐6, IL‐8 and IL‐10 in BALF of mice. The measurement data were expressed by the mean ± standard deviation. The comparison between the two groups was analysed by an unpaired *t* test. The experiment was repeated 3 times independently. * *P* < .05 vs. healthy donors. # *P* < .05 vs. normal mice. n = 10

Blood samples were firstly collected from healthy donors and patients with TRALI, as well as TRALI mouse models, to determine the level of expression of miR‐144. The results of the RT‐qPCR revealed that, compared with healthy human blood and normal mouse blood, the levels of miR‐144 in patients and mice with TRALI were significantly enhanced, respectively (*P* < .05) (Figure 1A,B). Moreover, the level of miR‐144 in lungs of mice with TRALI was shown by FISH assay to be up‐regulated (Figure 1C).

Since it has been suggested that activated platelets induce the formation of NETs in TRALI,^28^ we then examined whether NET was formed during TRALI through detection of the presence of MPO‐DNA, a degradation product of NET. According to the results (Figure 1D,E), the MPO‐DNA content in the blood samples of patients with TRALI was higher than that in blood samples of health donors (*P* < .05). Moreover, NET complexes in TRALI mice were markedly elevated, relative to that in normal mice. Besides, BALF collected from TRALI mice presented with decreased proportion of CD4^+^ cells (Figure 1F), accompanied by increased levels of IL‐6 and IL‐8 as well as decreased level of IL‐10 (Figure 1G). Collectively, these results indicated that miR‐144 was highly expressed in TRALI and that the activation of neutrophils was stimulated.

### 3.2 miR‐144 contributed to TRALI by promoting the formation of NET

**FIGURE 2:**
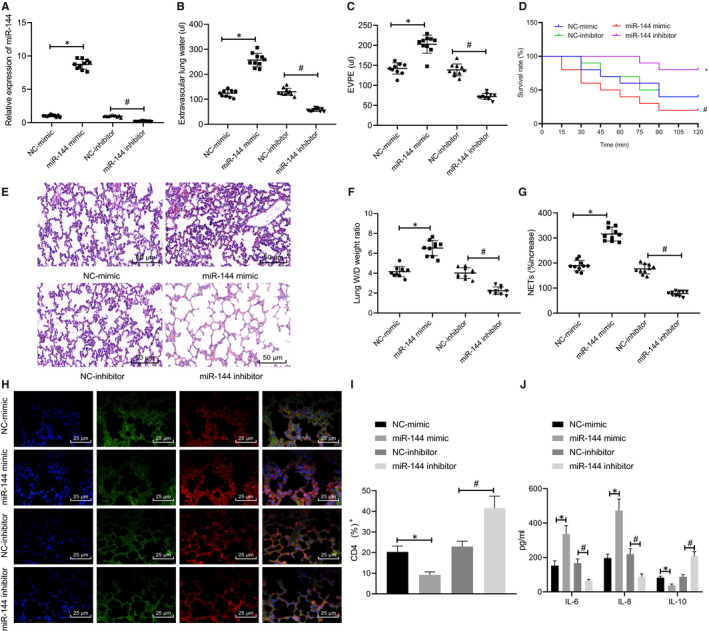
Down‐regulated miR‐144 alleviates TRALI by reducing the formation of NET. TRALI mice were treated with miR‐144 mimic/inhibitor. A, The expression of miR‐144 in the blood of TRALI mice from each group determined by RT‐qPCR. B, EVLW of mice in each group. C, EVPEs of mice in each group. D, Survival rate of mice within 2 h. E, The formation of NET in the blood of mice from each group measured by MPO‐DNA ELISA (×200). F, Lung injury identified by HE staining. G, Lung W/D ratios. H, The NET network structure in the lung tissues in each group of mice evaluated by immunofluorescence staining (×400). I, Flow cytometry to detect the proportion of CD4^+^ cells in BALF. J, ELISA to measure levels of IL‐6, IL‐8 and IL‐10 in BALF. The measurement data were expressed by the mean ± standard deviation. The experiment was repeated 3 times independently. Data among multiple groups were compared using a one‐way ANOVA, followed by Tukey's post hoc test. Kaplan‐Meier method was used to calculate the survival rate of mice, and survival difference was analysed by a log‐rank test. * *P* < .05 vs. NC mimic‐treated mice with TRALI. # *P* < .05 vs. NC inhibitor‐treated mice with TRALI. n = 10

miR‐144 was either inhibited or overexpressed in mice to explore the effects of aberrantly expressed miR‐144, which showed that the transfection of miR‐144 mimic up‐regulated the levels of miR‐144 in TRALI mice, while that of the miR‐144 inhibitor down‐regulated it (*P* < .05, Figure 2A).

The lung injury and survival time of modelled mice were also evaluated (Figure 2B‐D). Both EVLW and EVPEs were significantly elevated in mice treated with miR‐144 mimic, which might be the reason for the lowered survival rate (*P* < .05). Opposite results were induced by the treatment of the miR‐144 inhibitor. MPO‐DNA ELISA was later performed to detect the formation of NET in the blood of mice in each group (Figure 2E), of which the results revealed more NET complexes being presented in the blood of the mice in response to miR‐144 mimic treatment, while fewer of that resulted from miR‐144 inhibitor (*P* < .05). Afterwards, lung tissues were collected from mice in each group to identify lung injury by HE staining, measure lung W/D ratios and detect the network structure of NET by immunofluorescence staining. A large number of NETs were observed in lung tissue microstructures of mice treated with miR‐144 mimic, while the amount of NET was much smaller in mice treated with miR‐144 inhibitor, which might be accountable for the increased survival rate and improved lung injury outcome (Figure 2F‐H). Furthermore, the proportion of CD4^+^ cells in collected BALF was reduced in response to miR‐144 overexpression (*P* < .05) and increased in response to miR‐144 inhibition (Figure 2I). It was also observed that miR‐144 overexpression led to up‐regulated levels of IL‐6 and IL‐8 as well as down‐regulated level of IL‐10 in BALF and that miR‐144 inhibition led to the opposite (Figure 2J). In summary, the results demonstrated that the regulatory role of miR‐144 in TRALI may be dependent on the extent of the formation of NET.

### 3.3 miR‐144 targets KLF2 and down‐regulates its expression

**FIGURE 3:**
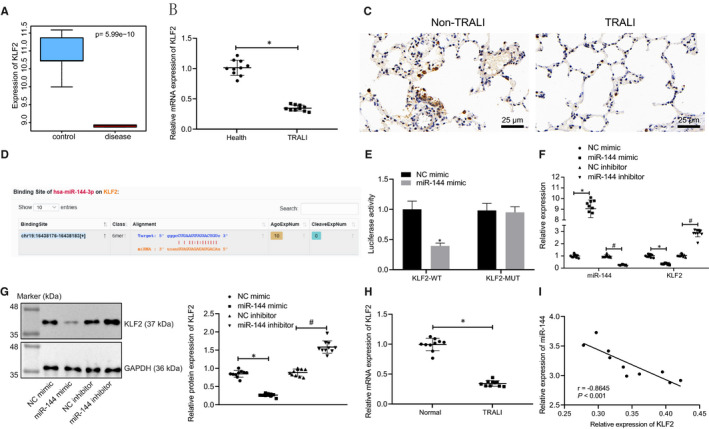
miR‐144 directly targets and negatively regulates KLF2. A, Box plots displaying the expression of KLF2 in normal samples (blue box on the left) and TRALI samples (red box on the right) from microarray data set GSE11434. B, The mRNA level of KLF2 in blood samples from healthy people and patients with TRALI determined by RT‐qPCR. C, Immunohistochemistry to detect the level of KLF2 in lung tissues of patients with TRALI (×400). D, The putative binding sites between miR‐144 and KLF2 predicted from the Starbase website. E, The binding relationship between miR‐144 and KLF2 verified by a dual‐luciferase reporter gene assay. F, The mRNA levels of miR‐144 and KLF2 in human blood samples after transfection of miR‐144 mimic/inhibitor measured by RT‐qPCR. G, KLF2 protein levels in human blood samples after transfection of miR‐144 mimic/inhibitor normalized to GAPDH evaluated by Western blot analysis. H, KLF2 mRNA levels in normal and TRALI mouse blood samples after transfection examined by RT‐qPCR. I, Pearson correlation analysis of the correlation between miR‐144 and KLF2 mRNA expression in mice. The measurement data were expressed by the mean ± standard deviation. The experiment was repeated 3 times independently. The comparison between the two groups was analysed by an unpaired *t* test. Data among multiple groups were compared using a one‐way ANOVA, followed by Tukey's post hoc test. Pearson correlation was used for correlation analysis. * *P* < .05 vs. healthy donors, the NC mimic group or NC mimic‐treated mice with TRALI. # *P* < .05 vs. NC inhibitor‐treated mice with TRALI. n = 10

A previous study has shown that KLF2 is associated with ALI in various animal models.^11^ KLF2 was found to be down‐regulated (Figure 3A), as depicted in the box plot from the data in GSE11434. The mRNA level of KLF2 in blood samples of healthy donors and patients with TRALI was determined by RT‐qPCR (Figure 3B). The expression of KLF2 in blood samples of patients with TRALI was significantly reduced (*P* < .05) when compared with that of healthy donors, thus indicating the down‐regulation of KLF2 in TRALI. KLF2 level in the lung tissues of TRALI patients was also revealed to be reduced (Figure 3C).

The bioinformatics prediction website (Starbase) indicated that there is the presence of a binding relationship between miR‐144 and KLF2 (Figure 3D), which was then verified by a dual‐luciferase reporter gene assay (Figure 3E). The miR‐144 mimic significantly reduced the luciferase activity in the KLF2‐WT group (*P* < .05) when compared with that of the NC mimic, while the luciferase activity in the KLF2‐MUT group revealed no significant changes (*P* > .05). After the transfection of miR‐144 mimic or miR‐144 inhibitor, the mRNA and protein levels of KLF2 in blood samples of patients with TRALI and TRALI mice were evaluated through RT‐qPCR and Western blot analysis. As shown in Figure 3F‐H, Supplementary Figure 1A, miR‐144 mimic treatment down‐regulated the expression of KLF2 significantly, but miR‐144 inhibitor led to opposite trends (*P* < .05). Further correlation analysis revealed an inverse relationship between miR‐144 and KLF2 (Figure 3I). In brief, miR‐144 could target KLF2 by negatively regulating its expression.

### 3.4 miR‐144 promoted NET‐induced TRALI by reducing KLF2

**FIGURE 4:**
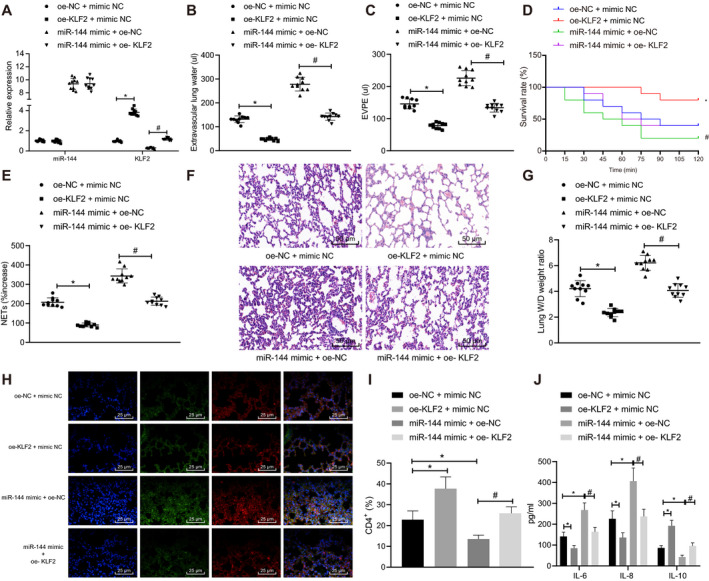
miR‐144 targets KLF2 to aggravate NET‐induced TRALI. TRALI mice were treated with oe‐KLF2, miR‐144 mimic or oe‐KLF2 + miR‐144 mimic. A, The expression of miR‐144 and KLF2 in blood samples of mice after transfection measured by RT‐qPCR. B, EVLW of mice in each group. C, EVPEs of mice in each group. D, Survival rate of mice in each group within 2 h. E, The formation of NET in the blood of mice in each group detected by MPO‐DNA ELISA. F, Lung injury identified by HE staining (× 200). G, Lung W/D ratios. H, The NET network structure of the lung tissues of mice in each group evaluated by immunofluorescence staining (×400). I, Flow cytometry to detect the proportion of CD4^+^ cells in BALF. J, ELISA to measure levels of IL‐6, IL‐8 and IL‐10 in BALF. The measurement data were expressed by the mean ± standard deviation. The experiment was repeated 3 times independently. The data comparison between the two groups was analysed by an unpaired *t* test. Data comparison among multiple groups was performed using a one‐way ANOVA, followed by Tukey's post hoc test. Kaplan‐Meier method was used to calculate the survival rate of mice, a log‐rank test was used to test the difference of survival, and the relationship between X and Y was analysed by Pearson correlation. * *P* < .05 vs. oe‐NC +mimic NC‐treated mice with TRALI. # *P* < .05 vs. miR‐144 mimic +oe‐NC‐treated mice with TRALI. n = 10

To investigate the regulatory role of KLF2 in TRALI, mice were subjected to oe‐KLF2 treatment (Supplementary Figure 1B). RT‐qPCR was then performed to determine the expressions of miR‐144 and KLF2 in mouse blood samples, which revealed that the oe‐KLF2 had no significant effects on the expression of miR‐144 (*P* > .05), but significant raised that of KLF2 (*P* < .05). Similar effects were observed when miR‐144 mimic and oe‐KLF2 were transfected simultaneously in comparison with the treatment of miR‐144 mimic alone (Figure 4A).

The lung injury and survival rate were studied (Figure 4B‐D). In response to overexpressed KLF2, both EVLW and EVPEs were significantly decreased, while the survival rate increased (*P* < .05). Similarly, both EVLW and EVPEs were noticeably elevated, accompanied by higher survival rate, in response to the simultaneous overexpression of miR‐144 and KLF2 as compared with miR‐144 overexpression alone (*P* < .05).

MPO‐DNA ELISA was subsequently conducted to detect the formation of NET in the blood of mice in each group (Figure 4E). The up‐regulation of KLF2 evidently increased the content of NET complexes. Also, the co‐treatment of miR‐144 mimic +oe‐KLF2 reduced NET complexes compared with that of miR‐144 mimic +oe‐NC (*P* < .05).

Moreover, lung injury was detected by HE staining, lung W/D ratios were measured, and NET network structure was determined by immunofluorescence staining (Figure 4F‐H). A large number of NET complexes had existed in the lung microstructure of mice treated with oe‐NC +mimic NC or mice treated with miR‐144 mimic +oe‐NC. The number of NET structure was increased in response to miR‐144 up‐regulation but decreased in response to KLF2 up‐regulation.

Flow cytometry detection of BALF then unravelled reduced proportion of CD4^+^ cells in BALF in the presence of miR‐144 overexpression but increased proportion of them in the presence of KLF2 overexpression, and the simultaneous overexpression of miR‐144 and KLF2 could reverse miR‐144 mimic‐induced decrease in the number of CD4^+^ cells (Figure 4I). Consistently, miR‐144 overexpression up‐regulated IL‐6 and IL‐8 and down‐regulated IL‐10, while KLF2 overexpression led to the opposite, and additional KLF2 overexpression abrogated the effects of miR‐144 overexpression alone (Figure 4J).

Taken together, the aforementioned results demonstrated that overexpressed KLF2 alleviated NET‐induced TRALI and even reversed the stimulative effects of miR‐144 overexpression on TRALI. In other words, miR‐144 could inversely regulate KLF2 to promote the formation of NET and further aggravate TRALI.

### 3.5 miR‐144 inhibited KLF2 to activate the NF‐κB/CXCR1 signalling pathway, thereby aggravating NET‐induced TRALI

**FIGURE 5:**
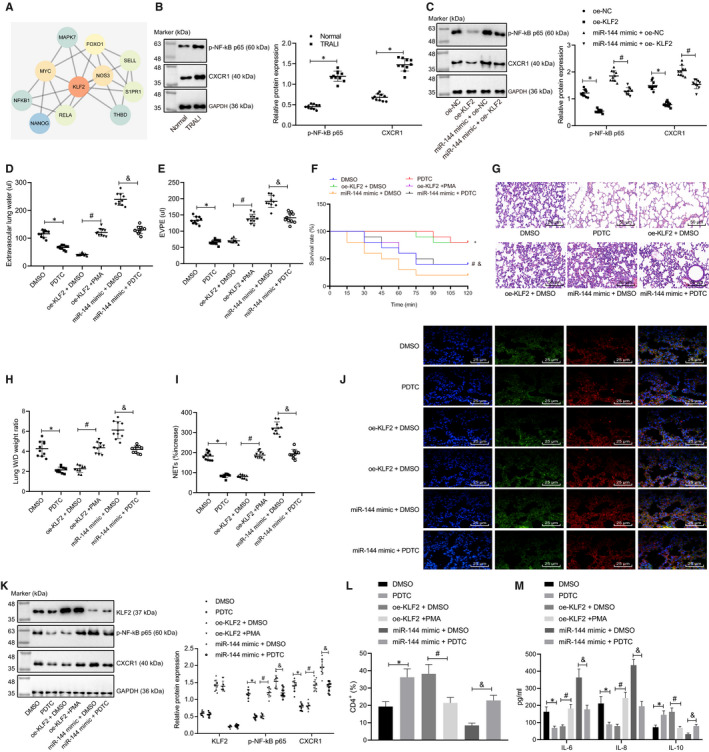
miR‐144 inhibits KLF2 to activate the NF‐κB/CXCR1 signalling pathway, thereby aggravating NET‐induced TRALI. A, A PPI network diagram of KLF2 and its related genes constructed by the online analysis tool String. The deeper red colour of the circle indicates the higher core degree, while the deeper blue colour indicates the lower core degree. B, The extent of NF‐κB p65 phosphorylation and CXCR1 protein level normalized to GAPDH in neutrophils of normal and TRALI mice evaluated by Western blot analysis. C, The extent of NF‐κB p65 phosphorylation and CXCR1 protein level normalized to GAPDH in neutrophils of TRALI mice treated with oe‐KLF2, miR‐144 mimic or oe‐KLF2 + miR‐144 mimic evaluated by Western blot analysis. TRALI mice were then treated with DMSO, PDTC, oe‐KLF2 + DMSO, oe‐KLF2 + PMA, miR‐144 mimic +DMSO or miR‐144 mimic +PDTC. D, EVLW of TRALI mice in each group. E, EVPEs of TRALI mice in each group. F, Survival rate of TRALI mice in each group within 2 h. G, Lung injury identified by HE staining (×200). H, Lung W/D ratios. I, The formation of NET in the blood of TRALI mice in each group detected by MPO‐DNA ELISA. J, The NET network structure of the lung tissues of TRALI mice in each group measured by immunofluorescence staining (×400). K, The extent of NF‐κB p65 phosphorylation and protein expression levels of KLF2 and CXCR1 normalized to GAPDH in neutrophils of TRALI mice in each group tested by Western blot analysis. L, Flow cytometry to detect the proportion of CD4^+^ cells in BALF. M, ELISA to measure levels of IL‐6, IL‐8 and IL‐10 in BALF. The measurement data were expressed by the mean ±standard deviation. The experiment was repeated 3 times independently. The data comparison between the two groups was conducted by an unpaired *t* test. The data comparison among multiple groups was performed using a one‐way ANOVA, followed by Tukey's post hoc test. Kaplan‐Meier method was used to calculate the survival rate of mice, and a log‐rank test was used to test the difference regarding survival. * P < .05 vs. normal mice, oe‐NC‐ or DMSO‐treated mice with TRALI. # *P* < .05 vs. miR‐144 mimic +oe‐NC‐ or oe‐KLF2 + DMSO‐treated mice with TRALI. & *P* < .05 vs. miR‐144 mimic +DMSO‐treated mice with TRALI. n = 10

A PPI network of KLF2 and its related genes was constructed, and 10 related genes were obtained with the STRING function protein association network (Figure 5A). Previous researches have shown that NF‐κB1, one of the critical genes implicated in the NF‐κB signalling pathway, plays an important role in ALI.^15^, ^16^, ^17^ NF‐κB can also mediate the expression of CXCR1,^18^ and is a potential therapeutic target for ALI.^19^, ^20^, ^21^ Therefore, a hypothesis was proposed that KLF2 might inhibit the transcriptional activity of NF‐κB to affect the NET‐induced TRALI. The protein expression levels of NF‐κB p65 and CXCR1 in neutrophils of normal mice and TRALI mice were determined by Western blot analysis (Figure 5B). The results revealed that the extent of NF‐κB p65 phosphorylation and the level of CXCR1 in TRALI mice were significantly increased (*P* < .05), indicating that the NF‐κB signalling pathway was activated in TRALI. Subsequently, in response to the treatment of oe‐KLF2, the reduced levels of NF‐κB p65 phosphorylation and CXCR1 were observed (*P* < .05). Besides, the addition of miR‐144 mimic still maintained the current trends of the obtained results (*P* < .05) (Figure 5C).

Based on these results, we speculated that KLF2 might inhibit the activation of the NF‐κB signalling pathway, thereby alleviating the formation of NET and TRALI.

The NF‐κB inhibitor (PDTC) and NF‐κB activator (PMA) were used to treat TRALI mice in the presence of oe‐KLF2 or miR‐144 mimic. The resulting EVLW, EVPEs, survival rate of mice, lung injury and lung W/D ratio were later analysed (Figure 5D‐H). It was observed that when the NF‐κB signalling pathway was inactivated by PDTC, both EVLW and EVPEs were significantly reduced, thus resulting in an increased survival rate (*P* < .05). Once the NF‐κB signalling pathway was activated by PMA with the presence of oe‐KLF2, both EVLW and EVPEs were significantly increased, resulting in a lower survival rate (*P* < .05). The treatment with miR‐144 mimic +PDTC not only evidently decreased EVLW and EVPEs, but also up‐regulated the survival rate of TRALI mice, when compared with that of the treatment with miR‐144 mimic +DMSO.

MPO‐DNA ELISA and immunofluorescence staining were carried out next to detect the formation of NET complexes in the blood of mice and to also evaluate the NET structure (Figure 5I,J). The results indicated that the PDTC treatment significantly down‐regulated the amount of NET complexes in the blood and NET network in mouse lung microstructure, compared with that of the DMSO treatment (*P* < .05). On the contrary, an uptrend was observed in mice treated with PMA once oe‐KLF2 plasmids were added (*P* < .05). In relative to mice treated with miR‐144 mimic +DMSO, the co‐treatment with miR‐144 mimic +PDTC resulted in reduced NET complexes and NET network (*P* < .05).

The protein levels of KLF2, NF‐κB p65 and CXCR1 in neutrophils of TRALI mice were also detected at the same time (Figure 5K). There was no significant difference regarding the expression of KLF2 protein in neutrophils of mice that were treated with DMSO or PDTC (*P* > .05), while the extent of NF‐κB p65 phosphorylation and CXCR1 was significantly reduced after PDTC treatment, compared with that of DMSO (*P* < .05). In comparison with the treatment of oe‐KLF2 + DMSO, the expression levels of KLF2 and CXCR1, as well as the extent of NF‐κB p65 phosphorylation, were significantly heightened after the treatment of oe‐KLF2 + PMA (*P* < .05). No significant changes of KLF2 protein levels were witnessed in mice treated with miR‐144 mimic +PDTC, when compared with that of mice treated with miR‐144 mimic +DMSO (*P* > .05). However, the extent of NF‐κB p65 phosphorylation and CXCR1 protein level was down‐regulated following the treatment of miR‐144 mimic +PDTC (*P* < .05).

In addition, PDTC treatment resulted in increased proportion of CD4^+^ cells, down‐regulated IL‐6 and IL‐8 and up‐regulated IL‐10 in BALF as compared with DMSO, either with or without the presence of miR‐144 overexpression; and BALF from mice of the oe‐KLF2 + PMA group presented with reduced CD4^+^ cells relative to those of the oe‐KLF2 + DMSO group (Figure 5L,M). Based on the aforementioned results, it was concluded that miR‐144 inhibited the expression of KLF2 to activate the NF‐κB signalling pathway, thereby facilitating the progress of NET‐induced TRALI.

## 4 DISCUSSION

**FIGURE 6:**
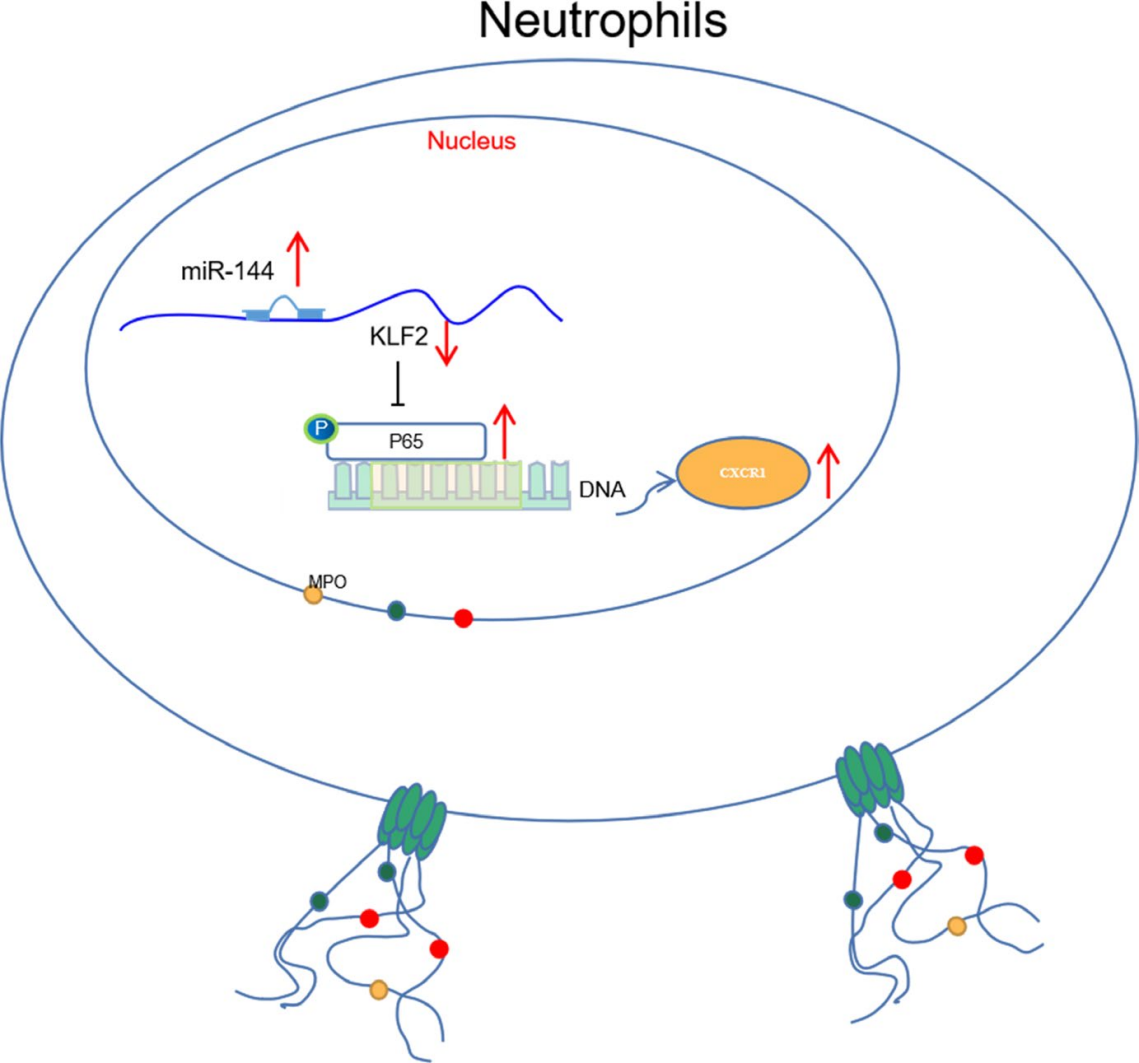
Mechanical graph of the role of miR‐144 in TRALI. Up‐regulated miR‐144 promotes the formation of NET through activating the NF‐κB/CXCR1 signalling pathway *via* down‐regulation of KLF2, thus exacerbating TRALI

The molecular mechanisms underlying lung injury have not been thoroughly investigated yet^29^ especially in the field of ALI.^30^ Herein, the present study investigated the mechanism by which miR‐144 mediated TRALI. Our study demonstrated that miR‐144 contributed to the progression of NET‐induced TRALI by activating the NF‐κB/CXCR1 signalling pathway *via* KLF2 (Figure 6).

MiRNAs, single‐stranded non‐coding RNAs consisting of an average of 22 nucleotides, play a crucial biological regulatory role in multiple pathophysiological processes. For example, miRNA expression is found to be abnormal during the onset of ALI.^31^ In this study, we identified a significant increase in the expression of miR‐144 in TRALI. Similarly, a previous study has demonstrated the presence of highly expressed miR‐144 in ALI.^32^ Additionally, one report focusing on miRNA expression pattern in nanosized SiO‐induced lung damage was found to be accompanied by the up‐regulation of miR‐144.^33^

Furthermore, miRNAs can regulate an expression of a target gene at the transcriptional and post‐transcriptional level by binding to the target mRNA part and participating in the entire pathogenesis of ALI. For instance, Hu K *et al* have reported that miR‐1246 suppresses ALI‐induced inflammation and apoptosis via the NF‐κB and Wnt/β‐catenin signalling pathways.^34^ In the present study, the downstream targets of miR‐144 were studied. The website of Starbase indicated that miR‐144 and KLF2 had a targeted binding relationship and were poorly expressed in TRALI, which was further verified by a dual‐luciferase reporter gene assay. Some other downstream genes of miR‐144 have been notably identified to be involved in the action of miR‐144 in the lung, including Rho‐associated kinase‐1, Aquaporin‐1 and nuclear factor (erythroid‐derived 2)–like 2,^35^ suggesting the warrant of future investigations to rule out the interference in the clinic. Flow cytometry and ELISA analyses of the current study unravelled reduced proportion of CD4^+^ cells in BALF and elevated inflammatory cytokine production in the presence of miR‐144 overexpression but increased proportion of them and restricted inflammatory cytokine production in the presence of KLF2 overexpression. In line with our results, experimental lung injury has been elucidated to reduce the expression of KLF2 to increase endothelial permeability via the regulation of RAPGEF3‐Rac1 signalling.^14^ Moreover, the down‐regulation of KLF2 has been demonstrated in alveolar macrophages from rats with LPS‐induced ALI.^36^ Also, reduced expression of KLF2 has been elucidated during pathogenesis of paraquat‐induced ALI, functioning as a diagnostic marker at the early stage.^37^

NETs, detected in alveoli during TRALI, have been suggested to injure tissues and facilitate lung endothelial injury; NET formation has also been proposed as a potential approach to the therapy of TRALI.^28^, ^38^ In order to further study the mechanism of miR‐144 targeted inhibition of KLF2 on neutrophil chemotactic formation of NET, a PPI network of KLF2 and its related genes was constructed by the STRING online protein association network. It was proved that miR‐144 inhibited the expression of KLF2, thereby activating the NF‐κB/CXCR1 signalling pathway and promoting the formation of NET, thus contributing to the progression of TRALI. The formation of NETs has been suggested to be accompanied by the activation of NF‐κB signalling pathway in macrophages, whereas the marginal induction of CXCR1 has been observed in circulating neutrophils recruited by the lung.^39^, ^40^ Largely in agreement with our findings, miR‐326 activates the NF‐κB signalling pathway through target inhibition of B cell leukaemia/lymphoma 2–related protein A1, by which inflammatory responses and lung injuries of mice with ALI were aggravated.^41^ More importantly, function studies have shown that the inactivation of the NF‐κB signalling pathway can reduce oxidative stress to allow improvement of ALI; CXCR1/2 antagonists can ameliorate LPS‐induced ALI in association with sepsis.^42^, ^43^ Concordantly, the addition of PDTC, an inhibitor of the NF‐κB signalling pathway, resulted in alleviated lung injuries induced by NET, as observed in mouse models of TRALI in our study.

## 5 CONCLUSIONS

In summary, we found that highly expressed miR‐144 promoted the formation of NET through the NF‐κB signalling pathway *via* down‐regulating KLF2, thereby contributing to the pathophysiological process of TRALI. The present study deepened our understanding of TRALI pathogenesis and provided novel insights into for the treatment of TRALI. Nevertheless, the clinical evaluation of miR‐144 inhibitor as a treatment agent for TRALI still warrants further validation.

## CONFLICT OF INTEREST

The authors declare no conflict of interests.

## AUTHOR CONTRIBUTIONS

**Aiping Le:** Conceptualization (lead); Data curation (equal); Formal analysis (equal); Funding acquisition (lead); Investigation (lead); Methodology (lead); Writing‐original draft (lead); Writing‐review & editing (equal). **Yize Wu:** Conceptualization (lead); Data curation (equal); Formal analysis (equal); Investigation (lead); Methodology (lead); Writing‐original draft (lead); Writing‐review & editing (equal). **Wei Liu:** Data curation (equal); Formal analysis (equal); Project administration (equal); Resources (equal); Software (equal); Writing‐original draft (supporting); Writing‐review & editing (equal). **Chenggao Wu:** Data curation (equal); Formal analysis (equal); Project administration (supporting); Resources (equal); Software (equal); Writing‐review & editing (equal). **Piaoping Hu:** Supervision (equal); Validation (equal); Visualization (equal); Writing‐review & editing (equal). **Juan Zou:** Supervision (equal); Validation (equal); Visualization (equal); Writing‐review & editing (equal). **Linju Kuang:** Supervision (equal); Validation (equal); Visualization (equal); Writing‐review & editing (equal).

## Supporting information

Fig S1

Table S1

## Data Availability

The data sets generated for this study are available on request to the corresponding author.
