# Supplementary figures and images for: MiR‐144‐induced KLF2 inhibition and NF‐kappaB/CXCR1 activation promote neutrophil extracellular trap–induced transfusion‐related acute lung injury
Source: J Cell Mol Med. 2021 Jun 13;25(14):6511–23. doi: 10.1111/jcmm.16650 (PMC8278117; doi:10.1111/jcmm.16650)

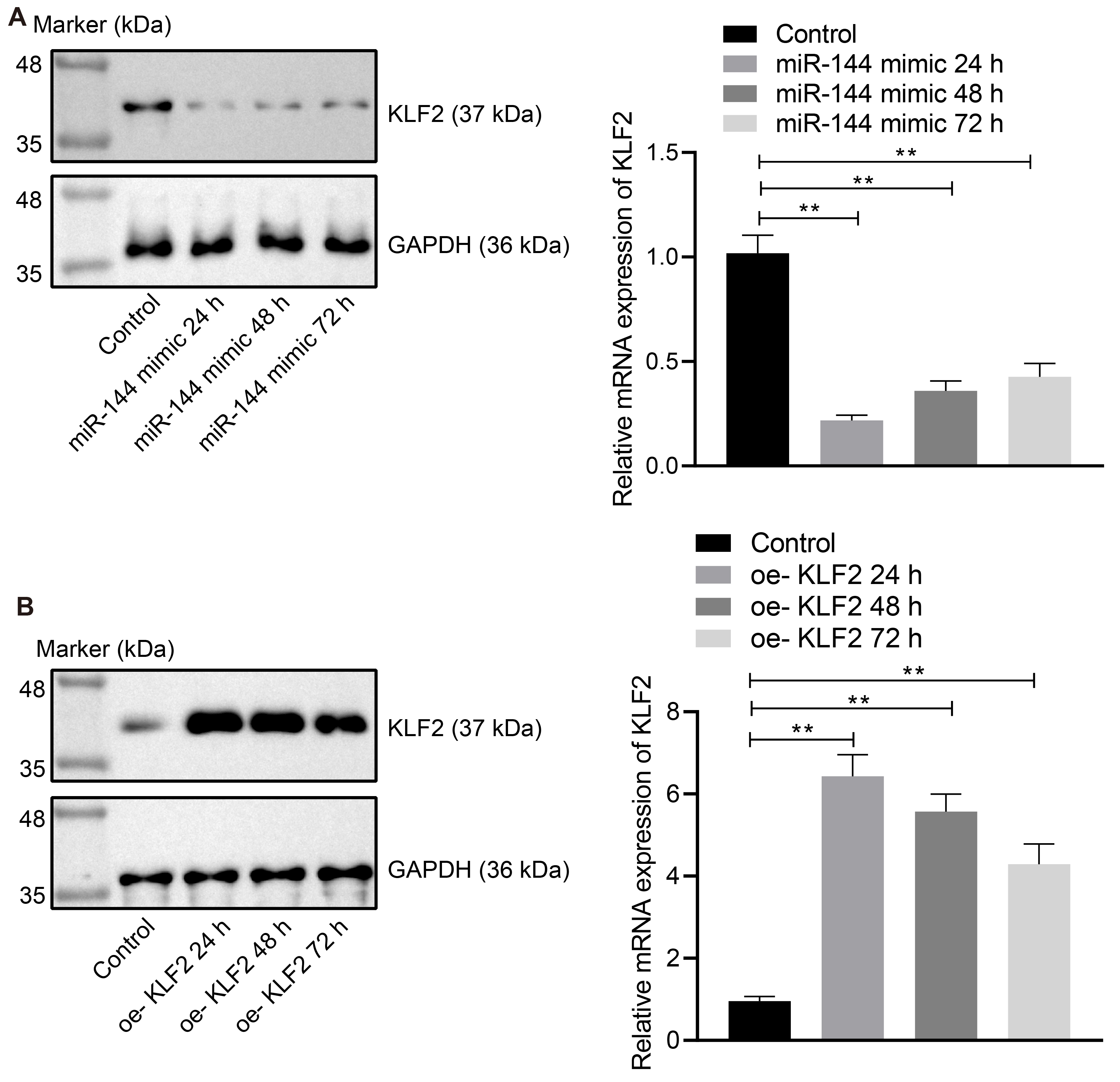

Supplement: Supplementary file 1 — Fig S1 [file JCMM-25-6511-s001.jpg]
