# Supplementary material for: MiR‐144‐induced KLF2 inhibition and NF‐kappaB/CXCR1 activation promote neutrophil extracellular trap–induced transfusion‐related acute lung injury
Source: J Cell Mol Med. 2021 Jun 13;25(14):6511–23. doi: 10.1111/jcmm.16650 (PMC8278117; doi:10.1111/jcmm.16650)
Supplement: Supplementary file 2 — Table S1 [file JCMM-25-6511-s002.docx]

**Supplementary table 1** Primer sequences for RT-qPCR

| Gene | Forward (5’-3’) | Reverse (5’-3’) |
| --- | --- | --- |
| miR-144 | GCTGGGATATCATCATATACTG | CGAGAATATCGATCTCATTC |
| KLF2 | AGCAGCATTGTACAGGGCTATGA | GCAGGGTCCGAGGTATTC |
| U6 | CTCGCTTCGGCAGCACA | AACGCTTCACGAATTTGCGT |
| GAPDH | GGAGCGAGATCCCTCCAAAAT | GGCTGTTGTCATACTTCTCATGG |

Note: RT-qPCR, reverse transcription quantitative polymerase chain reaction; miR-144, microRNA-144; KLF2, Krueppel-like factor 2; GAPDH, glyceraldehyde-3-phosphate dehydrogenase.
